# Circadian Regulation of Macrophages and Osteoclasts in Rheumatoid Arthritis

**DOI:** 10.3390/ijms241512307

**Published:** 2023-08-01

**Authors:** Nobuaki Kikyo

**Affiliations:** 1 Stem Cell Institute, University of Minnesota, Minneapolis, MN 55455, USA; kikyo001@umn.edu; 2 Department of Genetics, Cell Biology and Development, University of Minnesota, Minneapolis, MN 55455, USA

**Keywords:** circadian rhythms, cytokine, inflammation, macrophage, osteoclasts, rheumatoid arthritis

## Abstract

Rheumatoid arthritis (RA) represents one of the best examples of circadian fluctuations in disease severity. Patients with RA experience stiffness, pain, and swelling in afflicted joints in the early morning, which tends to become milder toward the afternoon. This has been primarily explained by the higher blood levels of pro-inflammatory hormones and cytokines, such as melatonin, TNFα, IL-1, and IL-6, in the early morning than in the afternoon as well as insufficient levels of anti-inflammatory cortisol, which rises later in the morning. Clinical importance of the circadian regulation of RA symptoms has been demonstrated by the effectiveness of time-of-day-dependent delivery of therapeutic agents in chronotherapy. The primary inflammatory site in RA is the synovium, where increased macrophages, T cells, and synovial fibroblasts play central roles by secreting pro-inflammatory cytokines, chemokines, and enzymes to stimulate each other, additional immune cells, and osteoclasts, ultimately leading to cartilage and bone erosion. Among these central players, macrophages have been one of the prime targets for the study of the link between circadian rhythms and inflammatory activities. Gene knockout experiments of various core circadian regulators have established that disruption of any core circadian regulators results in hyper- or hypoactivation of inflammatory responses by macrophages when challenged by lipopolysaccharide and bacteria. Although these stimulations are not directly linked to RA etiology, these findings serve as a foundation for further study by providing proof of principle. On the other hand, circadian regulation of osteoclasts, downstream effectors of macrophages, remain under-explored. Nonetheless, circadian expression of the inducers of osteoclastogenesis, such as TNFα, IL-1, and IL-6, as well as the knockout phenotypes of circadian regulators in osteoclasts suggest the significance of the circadian control of osteoclast activity in the pathogenesis of RA. More detailed mechanistic understanding of the circadian regulation of macrophages and osteoclasts in the afflicted joints could add novel local therapeutic options for RA.

## 1. Introduction

Rheumatoid arthritis (RA) is a chronic autoimmune disease characterized by joint swelling, stiffness, and pain that intensify in the early morning [1,2]. The signs and symptoms tend to become milder in the afternoon, although they could remain unchanged the whole day in severe cases of RA. The circadian fluctuations have been attributed to the changes in the serum concentrations of anti-inflammatory cortisol, pro-inflammatory melatonin, and pro-inflammatory cytokines including interleukin-1 (IL-1), IL-6, and tumor necrosis factor α (TNFα), among other factors [2,3,4,5,6,7]. These soluble factors regulate the activity of a variety of local immune cells, most importantly macrophages and T cells, and fibroblasts in the afflicted joints, forming a complex crosstalk among the cells. These fluctuations have been providing therapeutic opportunities based on time-of-day-dependent drug administration called chronotherapy, such as night-release of prednisone [7,8]. Chronotherapy has been primarily targeted toward the consequences of the circadian rhythmicity—fluctuating inflammations—rather than upstream controllers of rhythmicity. Circadian rhythmicity is orchestrated by a set of core regulators centered on the CLOCK/BMAL1 transcription factor complex. Although still in its infancy, direct targeting of the core regulators in the inflammatory cells in the afflicted joints can be alternative approaches. This review article first summarizes RA with an emphasis on the circadian regulations of the pathology. This is followed by discussions on the circadian control of macrophages and osteoclasts. The next chronotherapy section summarizes how the unique circadian features of RA can be exploited for therapeutic benefits. Finally, current limitations in the research and future directions are highlighted in the concluding remarks.

## 2. Overview of Rheumatoid Arthritis

The most prominent clinical features of RA are swelling, pain, bone erosion, and deformity in large and small joints, although cardiovascular complications are the primary causes of death [9,10,11,12,13]. RA affects 0.5–1% of U.S. adults, with a higher frequency in females than in males at a ratio of 2–3:1. While the precise etiology of RA remains unknown, a widely proposed hypothesis is as follows [9,12]. Environmental factors, such as microbiome, smoking, and chronic inflammation trigger post-translational modifications, including citrullination and carbamylation, of various proteins on the mucosal surface in the respiratory system, joints, or gut. The modified proteins are processed by dendritic cells and macrophages and subsequently presented as neo-antigens to T cells, which then activate B cells. Activated B cells then produce autoantibodies, most commonly anti-citrullinated protein antibodies (ACPAs, a widely used biomarker for RA). Genetic predisposition (>100 risk alleles, most famously HLA-DR) is also involved in these steps. These processes initiated by innate immunity elicit adaptive immunity through antigen-antibody complex formation, complement activation, and attraction of additional lymphocytes and other immune cells, leading to chronic arthritis. However, it is not clear why joints are predominantly affected by RA.

The primary inflammatory site in the joint is the synovium, where the synovial intimal lining (synovium surface facing the synovial fluid) shows hyperplasia, accompanied by abundantly infiltrated immune cells, predominantly macrophages and CD4^+^ T cells, but also B cells, neutrophils, and mast cells. The hyperplasia is caused by an increase in two types of cells in the intimal lining—macrophage-like synoviocytes (MLSs) and fibroblast-like synoviocytes (FLSs). MLSs are tissue-resident macrophages and developmentally distinct from circulating bone marrow-derived monocytes/macrophages (BMDMs) [13,14]. However, MLSs and BMDMs are closely related at the transcriptome level [15] and are not always clearly distinguished in the literature. Therefore, they are collectively called synovial macrophages hereafter. Synovial macrophages secrete a variety of pro-inflammatory cytokines, such as TNFα, IL-1, and IL-6, whereas FLSs produce proteases, including matrix metalloproteinases (MMPs) and cathepsin, in addition to cytokines. Complex networks between a variety of cells, cytokines, chemokines, and proteases lead to the erosion of the cartilage and bones mediated by FLSs and osteoclasts, and, ultimately, to joint deformities.

**Figure 1:**
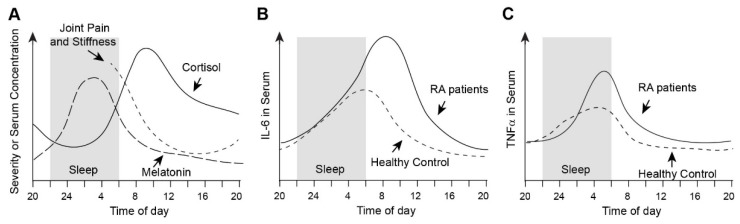
Summary of the circadian patterns of arthritis severity and the levels of hormones and cytokines involved in RA. (**A**) The levels of joint pain and stiffness in RA patients as well as the serum levels of cortisol and melatonin are shown. Cortisol and melatonin levels are similar in RA patients and healthy control. (**B**) Serum levels of IL-6 in RA patients and healthy control. (**C**) Serum levels of TNFα in RA patients and control. Although some reports could not detect circadian rhythmicity in the TNFα level, this graph represents an example of circadian expression in [2].

Circadian changes in the pathophysiology of RA have been extensively reviewed before [4,7,16,17]. The circadian patterns of joint pain and stiffness correlate with the circadian fluctuations in the serum levels of pro-inflammatory cytokines and various hormones. Although some variations are observed depending on the studies, a consensus is as follows. The joint symptoms are most severe early in the morning when the serum level of the pro-inflammatory melatonin is also high [2,7,18,19,20] (Figure 1A). The gradual decrease in the severity coincides with an increase in the serum level of the anti-inflammatory cortisol toward the late morning. The serum levels and patterns of melatonin and cortisol are similar between RA patients and healthy control, although the rhythmicity of the cortisol levels is diminished in some severe RA patients [2,21]. The serum level of one of the main cytokines for the arthritis IL-6 rises from midnight and reaches the peak level in the early morning in healthy control [2,5,22] (Figure 1B). However, the IL-6 level in RA patients keeps rising and reaches the highest level later in the morning. These patterns represent the clearest example of the difference between RA patients and control among several hormones and cytokines discussed here. Another central factor for arthritis TNFα does not show a consistent circadian expression pattern in RA patients (Figure 1C). While some reports found a higher circadian peak in the early morning in RA patients than in control [2], others did not detect any circadian oscillations in RA patients (healthy controls were not included in the studies) [5,22]. The discrepancy could be due the presence of various anti-inflammatory drugs in RA patients.

## 3. Roles of Macrophages in the Pathogenesis of RA

Synovial macrophages are one of the central players in joint inflammation in RA, along with T cell and FLSs [13,14,23]. First, synovial macrophages serve as antigen-presenting cells to T cells during the initial phase of RA, as mentioned above. A second, and more extensively studied, role is as a source of various cytokines (TNFα, IL-1, IL-6, IL-8, IL-10, IL-12, IL-15, IL-18, GM-CSF, M-CSF, and TGFβ) and chemokines (C-C- motif ligand 2 (CCL2), CCL3, CCL5, C-X-C motif ligand 1 (CXCL1), and CXCL8). These soluble factors often play multiple and overlapping roles in arthritis, forming a complex network. For example, TNFα and IL-1 are dominant pro-inflammatory cytokines in RA, sharing similar functions toward FLSs by stimulating their proliferation and secretion of various cytokines, reactive oxygen species, adhesion molecules, MMPs, cathepsin, and prostaglandins, which collectively lead to increased bone resorption. TNFα and IL-1 also modulate ion channels and increase pain transduction in sensory neurons [24]. Chemokines promote recruitment of neutrophils and synovial macrophages to inflammatory sites further exacerbating the inflammation. On the other hand, TGFβ plays anti-inflammatory roles by repressing T cell responses and downregulating the IL-1 receptor.

## 4. Outline of Mammalian Circadian Clocks

**Figure 2:**
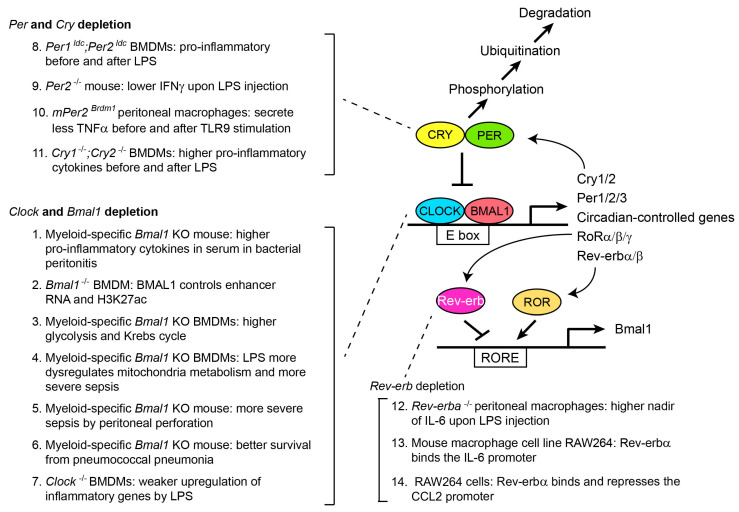
Summary of core circadian regulators and macrophage phenotypes after gene depletion. Phenotypes in comparison to wild-type mice or macrophages are described. See text for the details of the depletion phenotypes. Reference for each phenotype is as follows: 1 [27], 2 [28], 3 [29], 4 [30], 5 [31], 6 [32], 7 [33], 8 [34], 9 [35], 10 [36], 11 [37], 12 [38], 13 [39], and 14 [40].

Since this is a special issue dedicated to circadian rhythms, only a brief summary of mammalian circadian rhythms is described here, to provide sufficient information to understand the following discussion on macrophages and osteoclasts (Figure 2). Other articles in this issue (and recent review articles [25,26]) are recommended for more details. The CLOCK/BMAL1 heterodimer plays a central role in the regulation of mammalian circadian rhythms through binding the E-box (5′-CANNTG-3′) in promoters and enhancers of target genes, creating circadian oscillations of more than 20% of the genes in the genome in at least one tissue in the body. The activated target genes include the *Cry* (*Cry1* and *Cry2*) and *Per* (*Per1*–*Per3*) repressor genes. Translated CRY and PER proteins then bind the CLOCK/BMAL1 complex and inhibit the transcription activity, which forms the first negative feedback loop of the rhythms. However, CRY and PER gradually undergo phosphorylation and ubiquitination, leading to proteasomal degradation and release of CLOCK/BMAL1 from the inhibition, which completes the first feedback loop. In the second feedback loop, CLOCK/BMAL1 activates the genes encoding RORα retinoic acid receptor-related orphan receptor α RORβ and RORγ as well as Rev-erbα (reverse orientation c-erbAα) and Rev-erbβ. They bind the Rev-erb/ROR response elements (RREs) in the *Bmal1* promoter and activate (RORs) or inhibit (Rev-erbs) *Bmal1* transcription, forming the second feedback loop. Whereas these feedback loops are present in almost all tissues examined as peripheral clocks, they are synchronized by the central clock located in the suprachiasmatic nucleus in the hypothalamus, which is entrained by the light signal transmitted from the retina as the primary external cue (zeitgeber). Although the central clock synchronizes the peripheral clocks through autonomic nervous systems and hormones, the peripheral clocks are also independently entrained by various physiological factors such as body temperature, feeding time, and physical activity. Cultured cells also retain circadian rhythms in vitro and they can be readily synchronized by glucocorticoid, serum shock, forskolin, and other stimulations, serving as in vitro models for circadian regulation of cell physiology. This is particularly important for the study of human circadian rhythms due to the difficulty in repeatedly obtaining cells and tissues from the same person for experimental purposes.

## 5. Circadian Regulation of Inflammatory Macrophages

Several recent and comprehensive reviews are available on the circadian regulation of macrophage functions [41,42,43]. To begin with, around 8% of all genes show circadian oscillations in mouse peritoneal macrophages [44]. In another study, around 16% of the transcriptome showed circadian oscillation at the mRNA level, whereas 29% of the proteins were under circadian control in mouse BMDMs [45]. Importantly, 68% of oscillating mRNAs were not accompanied by protein oscillation and 86% of the oscillating proteins did not show oscillating mRNAs. This marked discrepancy between mRNA and protein levels underscores significant roles of circadian post-transcription regulation. The oscillating mRNAs and proteins encompass those involved in innate immunity, mitochondrial activity, and energy metabolism, which are all relevant to inflammation.

Circadian regulation of macrophages’ activity has been mainly studied as time-of-day-dependent differential responses to lipopolysaccharide (LPS) or Gram-negative bacteria in vivo or in vitro. As a member of the innate immune system, macrophages use the pattern recognition receptors (PRRs) on the surface to monitor infectious agents and tissue damage [42,46,47]. PRRs recognize microorganisms through pathogen-associated molecular patterns, such as LPS on the surface of Gram-negative bacteria, and damage-associated molecular patterns, including DNA and the chromatin protein HMGB1 released from damaged cells. LPS binds the PRR toll-like receptor 2 (TLR2) and TLR4, and activates the nuclear factor kappa B factor 1 (NF-κB), interferon regulatory factor 3 (IRF3), and activator protein 1 (AP-1) pathways, which leads to the activation of IL-1β, IL-6, TNFα, and IFN genes [48]. Thus, although LPS is unlikely to be directly relevant to RA in many cases, the downstream signaling pathways share many common features with those in synovial macrophages in RA.

The study of time-of-day-dependent immune responses goes back to a seminal work in 1960, when the authors reported a remarkable difference in the lethality of mice due to endotoxin shock upon intraperitoneal injection of LPS at different times of day [49]. When mice were entrained at 12 h light (Zeitgeber Time (ZT) 0-ZT12) and 12 h dark cycles (ZT12-ZT24), injection at ZT10 was 90% lethal, whereas the lethality was <15% when injected at ZT18. This model has been widely used to study the contributions of each circadian core regulator in the macrophages’ responses to LPS or bacterial injections, as described below.

The time-of-day-dependent inflammatory responses could be recaptured by harvesting splenic monocytes and macrophages every 4 h from mice and challenged by LPS in vitro [44]. Secretion of TNFα and IL-6 reached the peak levels when the cells were harvested from mice during ZT8-ZT12, corresponding to the most lethal time window. This time-of-day-dependent sensitivity appears to reflect oscillating gene expression at many levels of LPS-induced responses. The spectrum of the oscillating genes encompasses the components regulating the binding of LPS to TLR4, downstream mitogen-activated protein kinase (MAPK) pathway, NF-κB and AP-1 as critical transcription factors for pro-inflammatory cytokines, and regulators for the stability of cytokine mRNA.

## 6. Circadian Regulation of Macrophages by the CLOCK/BMAL1 Complex

The number of circulating monocytes demonstrates circadian fluctuations, which correlates with time-of-day-dependent sensitivity to infectious or inflammatory diseases. For example, the number of circulating pro-inflammatory Ly6C^hi^ monocytes was twofold higher at the peak between ZT4–ZT8 than at the nadir between ZT12–ZT20 in mice [27]. Reflecting the peak time, more Ly6C^hi^ monocytes were recruited to inflammatory sites when thioglycolate was intraperitoneally injected to induce peritonitis at ZT8 than at ZT0. Furthermore, in a bacterial peritonitis model induced by the injection of *Listeria monocytogenes*, which infects macrophages, an injection at ZT8 demonstrated fewer bacteria, more Ly6C^hi^ monocyte-derived dendritic cells, and higher levels of IL-1β, IL-6, IFNγ, and CCL2 in the peritoneum or peritoneal fluid than an injection at ZT0. However, when the same bacterial peritonitis was induced in myeloid-specific *Bmal1* knockout (KO) mice, survival rate was substantially decreased in both ZT0 and ZT8 injections. This was accompanied by elevated serum concentrations of IL-1β, IL-6, IFNγ, and CCL2, suggesting that BMAL1 represses inflammatory reactions (Ref. [36] in Figure 2). Indeed, BMAL1 recruits the polycomb repressive complex 2, which mediates the repressive H3K27me3 (trimethylation of lysine 27 in histone H3) mark to the regulatory elements in the *Ccl2* gene. This provides an example of BMAL1-mediated direct circadian inhibition of a pro-inflammatory gene through epigenetic regulation. BMAL1 also controls epigenetic landscape of pro-inflammatory genes through regulations of enhancer RNAs and the active enhancer mark H3K27ac (acetylation of lysine 27 in histone H3) [28].

Cell metabolism is another area that BMAL1 regulates during inflammation. BMDMs with myeloid lineage-specific *Bmal1* KO demonstrate enhanced glycolysis and the Krebs cycle activity, which generates more succinate, an inducer of IL-1β [29]. Challenge by LPS further dysregulates mitochondrial metabolism and increases production of reactive oxygen species, resulting in sepsis-induced tissue damage [30]. Additionally, BMAL1 regulates lactate metabolism, which is particularly important for lactate acidosis in septic shock [31]. This BMAL1-lactate axis controls T cell survivability in sepsis through production of the immune checkpoint protein PD-L1 by BMDMs. This appears to be one of the reasons why myeloid-specific *Bmal1* KO mice are more susceptible to sepsis induced by intestinal perforation. Thus, in these studies, *Bmal1*-depletion generally resulted in increased inflammation and higher lethality in mice upon injection of LPS or bacteria. However, myeloid-specific *Bmal1* KO protects mice from pneumococcal pneumonia, which was accompanied by increased cell motility and phagocytosis [32]. Therefore, a more reasonable conclusion would be that BMAL1 is necessary for the maintenance of proper macrophage activities during inflammation.

Much less information is available for the roles of CLOCK, a binding partner of BMAL1, in macrophages than for BMAL1. *Clock*^-/-^ BMDMs show weaker upregulation of inflammatory genes encoding IL-1β, IL-6, TNFα, IFNγ, and CCL2 in response to LPS compared with wild type (WT) cells in vitro [33]. Induction of IL-1β at the protein level is also reduced in the *Clock*^-/-^ cells compared with WT cells. These results are contradictory to the upregulation of the pro-inflammatory genes by *Bmal1* depletion in the several studies discussed above since CLOCK and BMAL1 are supposed to function as a heterodimer. The reason remains unclear; however, it is important to keep in mind that pro-inflammatory cytokines dysregulate the core circadian genes, forming bidirectional interactions between the circadian genes and inflammatory reactions, which further complicates the prediction of the consequences of the depletion of the circadian genes [17].

## 7. Circadian Regulation of Macrophages by PER, CRY, and Rev-erb

PER, CRY, and Rev-erb inhibit the CLOCK/BMAL1 complex, but their depletion generally hyperactivates macrophages, as in many studies of BMAL1 depletion, though with some exceptions. For example, BMDMs prepared from double mutant mice of *Per1* and *Per2* (*Per1^ldc^*;*Per2^ldc^*) were skewed toward the pro-inflammatory M1 status than to the anti-inflammatory M2 status without any stimulations compared with WT cells [34]. Additionally, once stimulated by LPS in vitro, the BMDMs more highly upregulated the *Tnfa* (encoding TNFα) and *Il1b* (encoding IL-1β) genes than WT cells. In contrast, two other studies demonstrated that a single depletion of *Per2* makes macrophages less pro-inflammatory. When *Per2*^-/-^ mice were injected with LPS in the first study, serum levels of TNFα and IL-1β were similar to those in WT mice, whereas the level of IFNγ was lower than in the WT mice due to defective production by NK and NKT cells [35]. Another study showed that peritoneal macrophages from *Per2* mutant mice (*mPer2^Brdm1^*) secreted less TNFα before and after stimulation by a TLR9 ligand compared with WT cells [36]. Myeloid-specific *Per* KO mice are required to understand more precise roles of PER-controlled circadian regulation of macrophages.

BMDMs from *Cry1*^-/-^;*Cry2*^-/-^ mice upregulate pro-inflammatory cytokine genes, such as *Il6*, *Cxcl1*, and *iNos*, compared with WT cells at the baseline [37]. When stimulated by LPS, the KO BMDMs secreted higher levels of TNFα and IL-6 than WT cells. Given the inhibitory role of CRY for the CLOCK/BMAL1 complex, this result looks unexpected. However, CRY does not function as a traditional transcription repressor of the CLOCK/BMAL1 complex in this case. In WT cells, CRY appears to bind and inhibit adenyl cyclase, which results in decreased cAMP and repressed protein kinase A (PKA) activity. However, in the absence of CRY, PKA is constitutively activated, leading to constitutive phosphorylation and activation of NF-κB and, finally, hyperactivation of pro-inflammatory genes.

Among several orthologues of ROR and Rev-erb, Rev-erbα has been the main target for the investigation. Time-of-day-dependent IL-6 secretion in response to LPS injection is lost in *Rev-erba*^-/-^ mouse macrophages [38]. Importantly, the cells retain general circadian rhythms, unlike *Bmal1*^-/-^*, Cry1*^-/-^;*Cry2*^-/-^, or *Per1*^-/-^;*Per2*^-/-^ cells, indicating a more specific link between *Rev-erba* and its target genes. Later, Sato et al. showed that Rev-erbα directly binds the RORE element in the *Il6* promoter, providing a mechanistic link between Rev-erbα and the response to LPS [39]. Rev-erbα also binds the RORE in the promoter of the *Ccl2* gene in the mouse macrophage cell line RAW264 and represses the expression [40]. This causes upregulation of the *Ccl2* gene in the *Rev-erbα^−/−^* peritoneal macrophages. The Rev-erbα-*Ccl2* connection could play an important role in inflammation, since CCL2 stimulates migration of the cells.

## 8. Osteoclastogenesis and Inflammation

Osteoclasts—only known bone resorbing cells—are directly responsible for the bone erosion and joint destruction in RA. Osteoclasts are multinucleated cells formed by the fusion of osteoclast progenitor cells derived from the monocyte/macrophage lineage although other cells, such as pre-dendritic cells and pre-B cells, can be transdifferentiated into osteoclasts [50,51,52]. M-CSF secreted by fibroblasts and receptor activator of nuclear factor-κB ligand (RANKL) produced by osteoblasts and osteocytes stimulate the differentiation of osteoclast progenitor cells to osteoclasts, including cell fusion and activation of genes involved in bone resorption. Osteoclasts use two mechanisms for bone resorption. First, they acidify and dissolve hydroxyapatite composed of calcium phosphate in the bone by secreting H^+^ and Cl^-^ ions. Second, they secrete proteases, such as cathepsin K (*Ctsk*), MMP9, and MMP14, that digest collagen I and other matrix proteins.

Osteoclastogenesis is promoted in inflammatory joints by various soluble factors, including RANKL, TNFα, IL-1, IL-6, and IL-17 secreted by FLSs, T cells, and B cells [53,54,55]. For example, RANKL is predominantly secreted by FLSs in a collagen-induced arthritis (CIA) model of the mouse [56], although it is also secreted by B cells [57]. RANKL secretion by FLSs is stimulated by IL-1, IL-6, and TNFα produced by macrophages, creating the macrophage–FLS–osteoclast axis for the RANKL-stimulated osteoclastogenesis in arthritis. In addition, TNFα, IL-1, and IL-6 stimulate osteoclastogenesis in RANKL-independent manners [55]. Since these cytokines are expressed in circadian manners, osteoclasts receive a network of external circadian cues for differentiation. Furthermore, they are under the intrinsic circadian control by the CLOCK/BMAL1 system as described below. Although definitive evidence is still missing, circadian control of osteoclastogenesis and bone resorption in RA patients is a likely possibility that awaits further investigation.

## 9. Circadian Regulation of Osteoclastogenesis

Circadian regulation of physiological osteogenesis and pathological bone resorption in osteoarthritis, osteoporosis, and other diseases has been extensively studied [58,59,60]. However, the roles of *Bmal1* in osteoclastogenesis remain unsettled, with contradictory studies reported by multiple groups. To start with an inhibition of osteoclastogenesis by *Bmal1* depletion, Xu et al. reported an increase in the bone mass in the femur and vertebrae in *Ctsk* promoter-driven osteoclast-specific *Bmal1* KO mice [61] (Table 1). Consistent with this phenotype, the KO mice demonstrated decreased osteoclast surface areas, osteoclast numbers, and the expression of osteoclast marker genes. Furthermore, in vitro differentiation by M-CSF and RANKL was disrupted in the *Bmal1* KO osteoclasts, as shown by diminished osteoclast numbers and bone resorption. These inhibitory effects might be explained by the lost activation of the nuclear factor of activated T cells 1 (*Nfatc1*) gene—the master regulator for osteoclastogenesis [62]—by BMAL1, which binds the E-box in the enhancer of the gene in a reporter assay with HEK293 cells. Supporting these findings, Fujihara et al. reported circadian expression of *Nfatc1* in the femur and the binding of BMAL1 to the same enhancer region in the macrophage cell line RAW264.7 [63].

In contrast, Zhao et al. found promoted osteoclast differentiation of RAW264.7 cells after *Bmal1* knockdown [64]. These opposing results of inhibition vs. promotion of osteoclastogenesis by *Bmal1* depletion could be due to the difference in the experimental conditions—cell types and depletion methods of *Bmal1*. Another contradictory study was reported by Tsang et al., who used *Ctsk* promoter-driven *Bmal1* KO mice, quite similar to the paper by the Xu group, but could not see any differences in the bone mass in the trabecular or cortical bone in the femur [65]. Both groups applied micro CT to the femur in 12-week-old mice. In addition, in vitro differentiation of osteoclasts was not affected by *Bmal1* KO in the Tsang paper, unlike in other works. To explain this discrepancy in the results, Tsang et al. listed several possibilities, such as the differences in genetic background of the mice and the stringency of using the littermate control, but the details remain to be determined.

*Bmal1*^-/-^ mice and *Clock*^-/-^ mice show diminished bone mass as cumulative effects on the opposing functions of osteoblasts and osteoclasts among many other cells, making the osteoclast-specific effects difficult to understand in general [66,67,68,69]. However, some of the studies successfully identified osteoclast-specific roles of *Bmal1* in the *Bmal1*^-/-^ mice. For example, *Bmal1* is necessary for the expression of osteoprotegerin (*Opg*), a decoy receptor of RANKL that inhibits osteoclastogenesis by preventing the genuine RANKL–RANK (the membrane receptor of RANKL) interactions [67,70]. Thus, in the absence of *Bmal1*, *Opg* is downregulated, resulting in increased osteoclasts and diminished bone mass. In another *Bmal1*^-/-^ mouse study, Takarada et al. found that the femur with decreased bone mass was accompanied by increased expression of bone resorption marker genes (e.g., *Tnfrsf11a* encoding RANK, *Tnfsf11* encoding RANKL, *Ctsk*, *Trap*, and *Mmp9*), while the expressions of the marker genes for osteoblasts and osteocytes were not altered [69]. In addition, by using an *Osx* promoter-driven osteoblast-specific *Bmal1* KO model, they demonstrated higher osteoclastogenesis induced by *Bmal1*-depleted osteoblasts, thus indirectly connecting the circadian regulator and osteoclastogenesis [69].

The roles of other circadian regulators in osteoclastogenesis are largely unknown. While bone mass in *Per1*^-/-^ mice and *Per2*^-/-^ mice was normal, it was increased in double disruption mice (e.g., in *Per1*^-/-^;*Per2^m/m^*, the *Per2* PAS [Per-Arnt-Sim] domain was deleted) in one study [71]. However, another study found that the disruption of the *Per2* PAS domain alone increased bone mass, depending on the mouse age [72,73]. Bone mass was also increased in *Cry1*^-/-^;*Cry2*^-/-^ mice and *Cry2*^-/-^ mice [71,72]. However, osteoclast-specific effects of the gene disruptions were not clear in these studies.

While these studies uncovered general roles of circadian core regulators in osteoclastogenesis, their significance in circadian RA symptoms is poorly understood. This is partly because of the difficulty in isolating osteoclasts from the affected joints at different times in a day. Additionally, osteoclast-specific KO mouse models have been only used for *Bmal1*. However, given the circadian expression of key cytokines for osteoclastogenesis (e.g., TNFα, IL-1, and IL-6), it would be surprising if osteoclast activity is immune from circadian fluctuations in the microenvironment.

**Table 1:** Bone and osteoclast phenotypes after *Bmal1* depletion.

Depletion Methods	Bone Mass	Osteoclasto-Genesis	References
*Ctsk* promoter-driven *Bmal1* KO	Increased	Decreased	[57]
*Bmal1* knockdown in RAW264.7 cells	Not applicable	Increased	[60]
*Ctsk* promoter-driven *Bmal1* KO	Normal	Normal	[61]
*Bmal1*^-/-^ (germline)	Decreased	Increased	[63]
*Bmal1*^-/-^ (germline)	Decreased	Increased	[65]
*Osx* promoter-driven *Bmal1* KO	Decreased	Increased	[65]

## 10. Chronotherapy

Since the specific causes of RA have not been elucidated, this disease is treated by a group of general immunomodulatory and anti-inflammatory drugs collectively called disease-modifying anti-rheumatic drugs (DMARDs), which can be divided into several categories. The oldest category, conventional synthetic DMARDs, includes the current first-line drug methotrexate (MTX) and other widely used drugs such as hydroxychloroquine and glucocorticoid [10,74]. Glucocorticoid is primarily used to control joint pain, stiffness, and swelling, rather than to slow down the progression of joint destruction in established RA [10,74,75]. It is most commonly used in the initial phase of the treatment and in the highly active phase of RA. However, due to many side effects, including hyperglycemia, infection, and osteoporosis, the dose is usually quickly tapered. Although conventional synthetic DMARDs are effective, the introduction of the second category, biologic DMARDs, in the form of TNFα inhibitors in the late 1990s has been hailed as a watershed moment in the history of the RA treatment [10,74,76,77]. The initial inhibitors were represented by Etanercept (a chimeric protein between TNFα receptor 2 and IgG Fc region, inhibiting the binding of TNFα to its receptor), approved by the U.S. Food and Drug Administration in 1998, and Infliximab (a chimeric anti-TNFα antibody blocking the binding of TNFα to its receptor), approved in 1999. To date, three additional TNFα antibodies have been approved for clinical use [10,74,78]. Furthermore, antibodies against IL-6 receptor, IL-1 receptor antagonist, antibodies against CD20 on B cells, and an inhibitor of T cell activation were subsequently developed, providing 10 biologic DMARDs in total. They have revolutionized the treatment of RA by greatly improving the inflammation and slowing the joint damage. However, it is also important to recognize the significant contributions to the revolution made by other changes that took place around the same time, such as early diagnosis and improved usage of MTX and folic acid [74]. In addition, since all biologic DMARDs modulate the immune system, they have a downside of increasing the risk of infection. The last category of DMARDs is small molecule DMARDs that include two Janus kinase (JAK) inhibitors [10]. They inhibit the IL-6 signaling pathway but do not block the IL-1 or TNFα pathway [79]. They are commonly used in non-responsive patients to biologic DMARDs.

As one of the oldest drugs for RA, glucocorticoids have been the most frequently applied drugs to chronotherapy among the DMARDs. One of the early studies found that administration of the synthetic corticosteroid prednisone at 22:00 was more effective than morning administration in reducing the morning stiffness in RA patients [80]. Subsequently, many studies verified that low dose administration of prednisone between 23:00–2:00 was more effective in decreasing morning stiffness and the serum concentration of IL-6 than the administration between 6:00–7:30 [7,8,81]. To overcome the inconvenience of taking a prednisone tablet at midnight, modified-release prednisone was later introduced to release prednisone 4 h after ingestion [75,82]. Two multicenter randomized controlled trials called CAPRA-1 and -2 (Circadian Administration of Prednisone in RA) demonstrated that taking modified-release prednisone at 22:00 and releasing it around 2:00–3:00 more effectively diminished morning stiffness duration, joint pain, fatigue, and the serum IL-6 level compared with talking immediate-release prednisone in the morning [83,84]. No clinically evident differences in adverse effects were observed between the modified-release and immediate-release prednisone in these studies. Additional clinical studies also validated the efficacy, safety, and cost-effectiveness of modified-release prednisone, despite the significantly higher costs of the modified-release prednisone compared with the immediate-release one [85,86,87]. Related to these findings, nonsteroidal anti-inflammatory drugs are more effective in improving RA symptoms when ingested at night or when delayed-release tablets targeting early morning are used, as demonstrated by several clinical studies [7].

**Figure 3:**
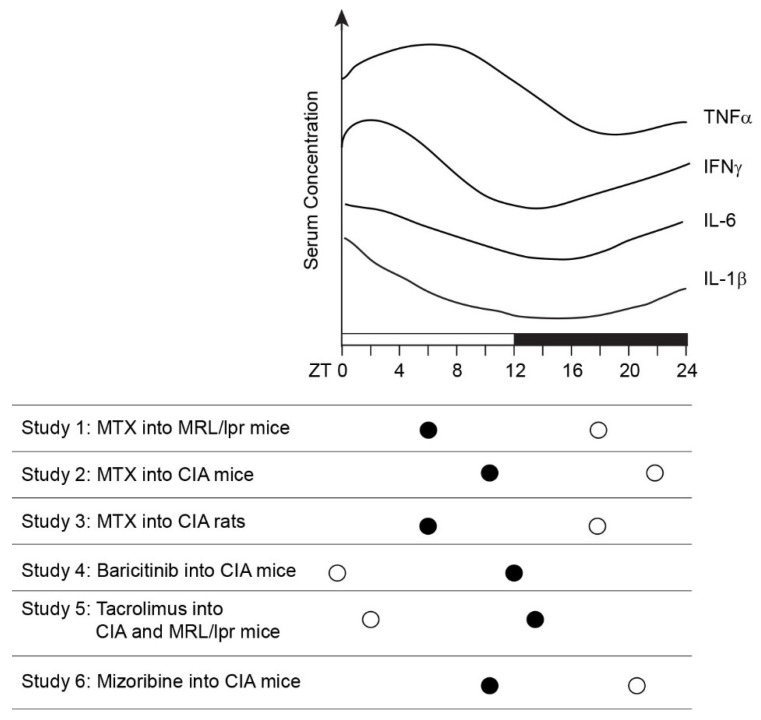
Summary of chronotherapy of mouse and rat arthritis models. The graph indicates serum concentrations of three inflammatory cytokines in the arthritis rodents [90,91,92]. Arthritis phenotypes were less severe when the indicated therapeutic agent was administered at the time shown by an open circle, compared with the administration time indicated by the closed circle. The reference for each study is as follows: Study 1 [89], Study 2 [94], Study 3 [93], Study 4 [92], Study 5 [91], and Study 6 [90].

Chronotherapy with other DMARDs has been reported with RA patients and mouse and rat arthritis models [8]. The mainstay in RA treatment MTX is a folate analogue with multiple anti-inflammatory mechanisms [88]. One of the dominant functions is to increase the intracellular concentration of adenosine, which suppresses IL-1, IL-6, and TNFα pathways. To et al. applied MTX to chronotherapy of a mouse arthritis model and human RA patients [89] (Figure 3, Study 1). They found that injection of MTX into MRL/lpr mice, a model for systemic autoimmunity including arthritis, at ZT18 decreased the serum levels of IgG-rheumatoid factor, TNFα, and serum amyloid A (SAA, a marker for acute inflammation) than the ZT6 injection [89]. The authors extended this finding to human RA patients in the same paper. In a single-arm clinical trial, the schedule of taking MTX was switched from three times per week (after breakfast and supper on day 1 and after breakfast on day 2) to once per day, three times per week at bedtime, without changing the total dose. After three months, the bedtime regimen improved joint swelling and tenderness, global patients’ activity, and serum markers for inflammation (C-reactive protein (CRP), SAA, IL-6, and TNFα) compared with the pre-switching regimen.

Six reports on the chronotherapy of mouse and rat arthritis models are summarized in Figure 3. In addition to MTX, Baricitinib (JAK1/3 inhibitor), Tacrolimus (immunosuppressant), and Mizoribine (immunosuppressant) have been used as therapeutic agents [89,90,91,92,93,94]. Their assessment parameters included the score of ankle joint swelling, histological analysis of ankle joints, and serum concentrations of IL-6, TNFα, and CRP. The results were surprisingly consistent: administration of these therapeutic agents while the inflammatory cytokine levels were rising or just past the peaks was more effective than the administration while the cytokine levels were declining. Considering the potential time-lag between the serum levels and the local inflammation intensity, this message suggests that these agents are more effective in preventing inflammation than in quenching full-blown inflammation. More direct inhibition of TNFα and IL-6 by biologic DMARDs could demonstrate clear benefits of chronotherapy in RA patients.

## 11. Concluding Remarks

In summary, a variety of experiments with depletion of each core circadian gene highlight the diversity and complexity of the mechanisms and functions of the circadian genes in inflammatory macrophages and osteoclasts. Bidirectional regulations between the circadian genes and pro-inflammatory cytokines further increase the complexity. Nonetheless, an overall conclusion would be that disruption of any core circadian regulators could hyper- or hypoactivate inflammatory responses by macrophages, mainly BMDMs and peritoneal macrophages in published cases. These acute and artificial inflammatory activations cannot be directly translated to the activation of synovial macrophages embedded in a complex web of cell–cell interactions over a long period of time in RA patients. However, given the difficulty of repeatedly obtaining enough synovial macrophages for research over the course of circadian rhythms, these non-synovial macrophages serve as more realistic materials to work with. In contrast, studies on circadian osteoclastogenesis in inflammatory environments, including RA, are by far left behind. Access to in vivo materials is one of the most important impediments.

The extensive studies on the roles of pro-inflammatory cytokines and downstream signaling pathways led to the development and clinical approval or trials of many biologic DMARDs. The unique temporal profiles of pro-inflammatory cytokines and hormones also led to chronotherapy of RA. On the other hand, targeting of the core circadian regulators remains in its infancy [95]. In addition to the complex roles mentioned above, multiple layers of post-translational regulations of the clock proteins are another reason for this situation. However, the spontaneous improvement in joint pain and swelling in the late afternoon is a powerful reminder that there is an under-exploited natural mechanism to diminish the symptoms, and that the core circadian regulators are the master controllers of arthritis. Although modulation of the circadian regulators would alter many physiological events in addition to inflammation, more detailed research on the roles of the circadian regulators in inflammation could lead to a novel type of drugs for RA.

## Abbreviations

ACTH	adrenocorticotropic hormone
ACPA	anti-citrullinated protein antibody
AP-1	activator protein 1
BMDM	bone marrow-derived monocyte/macrophage
CAPRA	Circadian Administration of Prednisone in RA
CIA	collagen-induced arthritis
Ctsk	cathepsin K
CCL2	C-C- motif ligand 2
CRP	C-reactive protein
CXCL1	C-X-C motif ligand 1
DMARD	disease-modifying anti-rheumatic drug
GM-CSF	granulocyte macrophage-colony stimulating factor
HALO	hours after the light was turned on
IFN	interferon
IFR3	interferon regulatory factor 3
IL-1	interleukin-1
FLS	fibroblast-like synoviocyte
H3K27ac	acetylation of lysine 27 in histone H3
H3K27me3	trimethylation of lysine 27 in histone H3
HMGB1	high mobility group box 1
JAK	Janus kinase
KO	knockout
LPS	lipopolysaccharide
MAPK	mitogen-activated protein kinase
M-CSF	macrophage-colony stimulating factor
MLS	macrophage-like synoviocyte
MMP	matrix metalloproteinase
MTX	methotrexate
Nfatc1	nuclear factor of activated T cells 1
NF-kB	nuclear factor kappa B subunit 1
NK cell	natural killer cell
NKT cell	natural killer T cell
Opg	osteoprotegerin
PAS	Per-Armt-Sim domain
PKA	protein kinase A
PRR	pattern recognition receptor
RA	rheumatoid arthritis
RANKL	receptor activator of nuclear factor-κB ligand
Rev-erbα	reverse orientation c-erbAα
RORα	retinoic acid receptor-related orphan receptor α
RRE	Rev-erb/ROR response element
SAA	serum amyloid A
TGFβ	transforming growth factor β
TLR2	toll-like receptor 2
WT	wild type
ZT	Zeitgeber Time
